# The Up-Regulation of Ribosomal Proteins Further Regulates Protein Expression Profile in Female *Schistosoma japonicum* after Pairing

**DOI:** 10.1371/journal.pone.0129626

**Published:** 2015-06-12

**Authors:** Jun Sun, Chen Li, Suwen Wang

**Affiliations:** Institute for Infectious Diseases and Vaccine Development, Tongji University School of Medicine, Shanghai, People’s Republic of China; Pohang University of Science and Technology, REPUBLIC OF KOREA

## Abstract

**Background:**

Pairing of *Schistosoma* males and females leads to and maintains female sexual maturation. However, the mechanism by which pairing facilitates sexual maturation of females is not clear. An increasing body of evidence suggests that ribosomal proteins have regulatory rather than constitutive roles in protein translation.

**Methodology/Principal Findings:**

To investigate the effect of ribosome regulation on female sex maturation, Solexa and iTRAQ techniques were used to analyze the relationship between ribosomal gene or protein expression and sexual development of *Schistosoma* females. In the present study, considerably higher number of ribosomal genes or proteins were found to be differentially expressed in paired 23-day-old females. Moreover, mature female-specific proteins associated with egg production, such as ferritin-1 heavy chain and superoxide dismutase, were selectively highly expressed in paired females, rather than higher level of protein synthesis of all transcripts compared with those in unpaired 23-day-old females. Furthermore, other developmental stages were utilized to investigate different expression pattern of ribosomal proteins in females by analysing 18-day-old female schistosomula from single- or double-sex infections to determine the relationship between ribosomal protein expression pattern and development. Results showed that undeveloped 18-day-old females from single- and double-sex infections, as well as 23-day-old unpaired females, possessed similar ribosomal protein expression patterns, which were distinct from those in 23-day-old paired females.

**Conclusions/Significance:**

Our findings reveal that the pairing of females and males triggers a specialized ribosomal protein expression profile which further regulates the protein profile for sexual maturation in *Schistosoma japonicum*, based on its gene expression profile.

## Introduction

The pairing of *Schistosoma* males and females in hosts, as well as their ultimate development into adult worms, is accompanied by remarkable morphological and molecular changes throughout their life cycle[1–5]. In particular, the pairing of males and females promotes and maintains female sexual maturation [6–8]. During this process, females need constant pairing contact with males to reach sexual maturation. Thus far, the exact mechanism by which pairing facilitates female development has not been investigated. Recent research revealed that females develop specific gene and miRNA profiles after pairing. This suggested that male contact might initiate the expression of specific miRNAs, which then regulate gene expression in a way that facilitates female sex maturation and egg production. Meanwhile, most ribosomal genes are upregulated after pairing, but most non-ribosomal genes are downregulated in paired females [9,10]. This phenomenon implies that ribosomal proteins serve an unusual role in female sexual maturation.

Ribosomes are highly conserved macromolecular machines responsible for protein synthesis in cells. Approximately 80 different ribosomal proteins are present in eukaryotic cells. These proteins are reported to regulate gene-specific transcription and translation processes [11]. Moreover, eukaryotic cells produce alternative ribosome variants to adapt to changing conditions [12]. Biochemical and proteomic data have shown that different ribosomal proteins were produced under different conditions [13–17]; that is, the ribosomal protein composition varies among tissues and developmental states [12,18,19]. Thus, the dynamic and heterogeneous expression patterns of ribosomal proteins probably have regulatory rather than constitutiveroles during translation.

In *Schistosoma japonicum* development, males and females begin to pair at about 18 days post-infection, and the female starts to lay eggs at about 24 dayspost-infection [20]. A previous study revealed that 23-day-old female schistosomula from double-sex infections (23DSI) and 23-day-old female schistosomula from single-sex infections (23SSI) or 18-day-old female schistosomula from double-sex infections (18DSI) exhibited different gene expression patterns[10]. This indicated that the pairing of *Schistosoma* females and males significantly changes gene expression in females. Consistent with this notion, diverse ribosome genes have been found to be significantly upregulated in mature females, suggesting that heterogeneous expression patterns of ribosomal proteins could be associated with the regulation of female development after pairing. To date, the role of ribosomal proteins in mediating female-specific changes after pairing is unclear. Results of the present study provide new insights into the role of ribosomal proteins in translational regulation during female sexual maturation and eggproduction.

## Material and Methods

### Ethics Statement

This study was carried out in strict accordance with the recommendations of the Regulations for the Administration of Affairs Concerning Experimental Animals of the State Science and Technology Commission. The protocol was approved by the Internal Review Board of Tongji University School of Medicine (TJmed-013-49).

### Unisexual and Paired Infections

Oncomelania hupensis snails were obtained from the Jiangsu Institute of Schistosome Diseases, Jiangsu province, China. Fifty female mice (Kunming strain) with a weight of 20 to 22 g were purchased from SLRC Laboratory Animal Co., Ltd. (Shanghai, China). Mice were kept under standard acclimatization conditions of 12 h light/dark cycle at 25°C and food and water were available ad libitum. To obtain single-sex female worms, the snails were exposed to a single miracidium to obtain single sex cercariae. Approximately 100 to 150 cercariae were used to percutaneously infect each mouse. Schistosomula were recovered by perfusion within 18 and 23 days post-infection, after mice were killed with an overdose of pentobarbital. To obtain double-sex female worms, about 100 to 150 multiple cercariae were used to percutaneously infect each mouse. The mice were sacrificed 18 days and 23 days post-infection, respectively. Females were recovered by washing with saline solution. The 23-day-old females were carefully separated from the paired worms under a microscope [2,9].

### Observation with Confocal Laser Scanning Microscopy (CLSM)

Different female samples were fixed in AFA (alcohol 95%, formalin 3%, and glacial acetic acid 2%) for at least 24 h, then stained with hydrochloric carmine for 30 min, and destained in acidic 70% ethanol until the worm turned light pink. After sequential dehydration in 70%, 80%, 90%, and 100% ethanol, for 5 min, respectively, worms were mounted on glass slides with neutral balsam. Images were acquired using a Leica TCS-SP5 Spectral Laser Scanning Confocal Microscope fitted with 488-nm He/Ne laser.

### Identification of Differentially Expressed Genes

Solexa was used to analyse whole genome gene expression in 23DSI and 23SSI, and 18DSI and 18SSI, as described previously[10]. Raw reads were filtered to obtain high quality data in the Tag-seq libraries of 23DSI and 23SSI, and 18DSI and 18SSI. All of the clean tags were mapped to the *S*. *japonicum* genome (http://www.chgc.sh.cn/japonicum/Resources.html) (predicted coding genes). The clean tags mapped to the reference sequences from multiple genes were filtered, and the remaining clean tags were designed and annotated as unambiguous clean tags. The initial counts of the clean tags of each gene were normalised (transcripts per million) to obtain normalised gene expression [21,22]. All differentially expressed genes were mapped to terms in the GO database to identify significantly enriched GO terms compared with the genome background, as previously described [10].

### Analysis of Differential Protein Expression Using iTRAQ and Mass Spectrometry

All reagents and buffers needed for iTRAQ labeling and cleaning were purchased from Applied Biosystems. The iTRAQ labeling assay was conducted as per the manufacturer’s instructions. Briefly, the proteins of different samples were respectively dissolved using 8 M Urea supplemented with 10 mM DTT, pH 8.5 and protein concentration was determined by the Bradford assay. Proteins were dissolved, denatured, alkylated, and digested with trypsin (1:20, w/w, 37°C for 18 h). To label peptides with iTRAQ reagents, 1 unit of label (defined as the amount of reagent required to label 100 μg of protein) was thawed and reconstituted in 70 μL ethanol. The digestions were labelled with 114 and 117 iTRAQ reagents, respectively. To identify more proteins, a strong cation exchange column was used for separating the mixed peptides. The following elution buffer was used: elution buffer A containing 5 mM K_2_HPO_4_ in 20% (v/v) acetonitrile, at pH 3.0 andelution buffer B containing 5 mM K_2_HPO_4_ in 20% (v/v) acetonitrile, 350 mMKCl at pH 3.0. The labelled peptides were reconstituted in phase A and injected at a flow rate of 0.7 mL/min into a high resolution strong cation exchange (SCX) column (4.6×250mm 5 μm), After loading, the SCX column and C 18 precolumn were flushed with a 3-step gradient sodium chloride solution (0, 50 and 100 mM) for 66 min. Afterward, the elute from the cation exchange column was collected with an Agilent 1100 series HPLC system equipped with an autosampler, 2 position six port valve, and diode array detector (220 nm) and 35 fractions were collected. Before LC-MS/MS, each fraction was desalted with a SP-10 column.

The eluted fractions were delivered into a nano RP column (5μm Hypersil C18, 75 μm × 150 mm) mounted in a Prominence Nano HPLC system, and were eluted with a 5%-40% ACN gradient containing 0.1% formic acid for 75 min at 400 nL/min. The eluates were directly entered into a triple quadrupole TOF 5600 System, fitted with a Nanospray III source and a pulled quartz tip as the emitter. The machine was run in positive ion mode and data-dependent manner with full MS scan from 350–1,800 m/z.

Data was acquired using an ion spray voltage of 2.5 kV, curtain gas of 30 PSI, nebulizer gas of 6 PSI, and an interface heater temperature of 150°C. The MS was operated with an RP of 30,000 FWHM for TOF-MS scans. For DDA, survey scans were acquired in 250 ms intervals and as many as 20 production scans were collected if exceeding a threshold of 125 counts per second (counts/s) and with a +2 to +5 charge-state. A Rolling collision energy setting was applied to all precursor ions for collision-induced dissociation. Dynamic exclusion was set for ½ of peak width (~8 s), and then the precursor was refreshed off of the exclusion list. Here, we used ProteinPilot software 4.0 to interpret raw data files produced by mass spectrometry. The parameters for searching were as follows: iTRAQ fourplex peptide labelled, trypsin digestion with only 1 maximum miss cleavage, carbamidomethylation for cysteine residue, variable oxidation for methionine, qTOF ESI, identification focus biological modifications. The tolerances were specified as ±0.05 Da for peptides and ±0.05 Da for MS/MS fragments. The NCBI database was chosen for searching and the FDR was controlled at 1% using the integrated tools in ProteinPilot. For protein assembling, the Pro Group algorithm was used to find the smallest number of proteins that could explain all the fragmentation spectral evidence. The data were analyzed with the R statistical software package (http://www.r-project.org).

### KEGG Pathway Analysis

Kegg pathway analysis of differentially-expressed genes was performed as described previously[10]. In addition, the studied differentially-expressed proteins were analyzed by blasting against Kegg genes (*Schistosoma*) to retrieve their KOs and were subsequently mapped to pathways in KEGG, as previously described [23].

### Quantitative RT–PCR Analysis

Several differentially expressed ribosomal genes, such as Sjc_0066600|Sjp_0066600|SJC_S000839.242218, Sjc_0000880|Sjp_0000880|SJC_S000001.1265306, Sjc_0044770|Sjp_0044770|SJC_S000379.21600 were selected for validation using quantitative RT–PCR. The analysis was performed as previously described [10]. Relative levels of gene expression were calculated using the 2^-ΔΔCT^ method [24]. Data are representative of at least 3 individual experiments.

### Western Blot

Proteins from 23DSI, 23SSI, 18DSI (or 18SSI) were separated on 12% polyacrylamide gels and transferred to PVDF membranes. These blots were incubated for 2h at room temperature in Tris-buffered-saline with Tween (20 mM Tris-Cl, 140 mM NaCl, pH 7.5, 0.05% Tween 20) containing 5% skim milk. Primary antibodies were anti-glyceraldehyde-3-phosphate dehydrogenase (GAPDH) monoclonal antibody (diluted 1:1000), RPL8 polyclonal antibody (diluted 1:1000), and RPL11 polyclonal antibody (diluted 1:1000). Blots were incubated with primary antibodies overnight at 4°C. After washing three times in Tris-buffered-saline with Tween, blots were incubated with horse radish peroxidase-conjugated secondary antibody (diluted 1:10,000) for 1h at room temperature. Immunoreactive complexes were visualized using ECL reagents.

### Motifidentification

The sequences of differentially expressed ribosomal genes were downloaded from here (http://www.chgc.sh.cn/japonicum/Resources.html). Then 1000bp upstream and 500bp downstream of the gene start sites were retrieved as the promoter sequence for each gene. MEME was applied to identify motifs in the promoter regions of the downregulated and upregulated ribosomal gene [25], using the following parameter settings: the distribution of motifs: zero or one occurrence per sequence; maximum number of motifs to find: 3. The motif must be present in the most members within the same group. Other options used the default values.

### Statistical Analysis

Results were presented as mean ± standard deviation from at least three independent experiments. Statistical analyses were performed using one-way ANOVA and Student’s t-test. A value of *P*< 0.05 was considered statistically significant.

## Results / Discussion

### Influence of Pairing on the Morphological Development of Female *S*. *japonicum* from Single- and Double-Sex Infection at 18 and 23 Days Post-Infection

**Fig 1:**
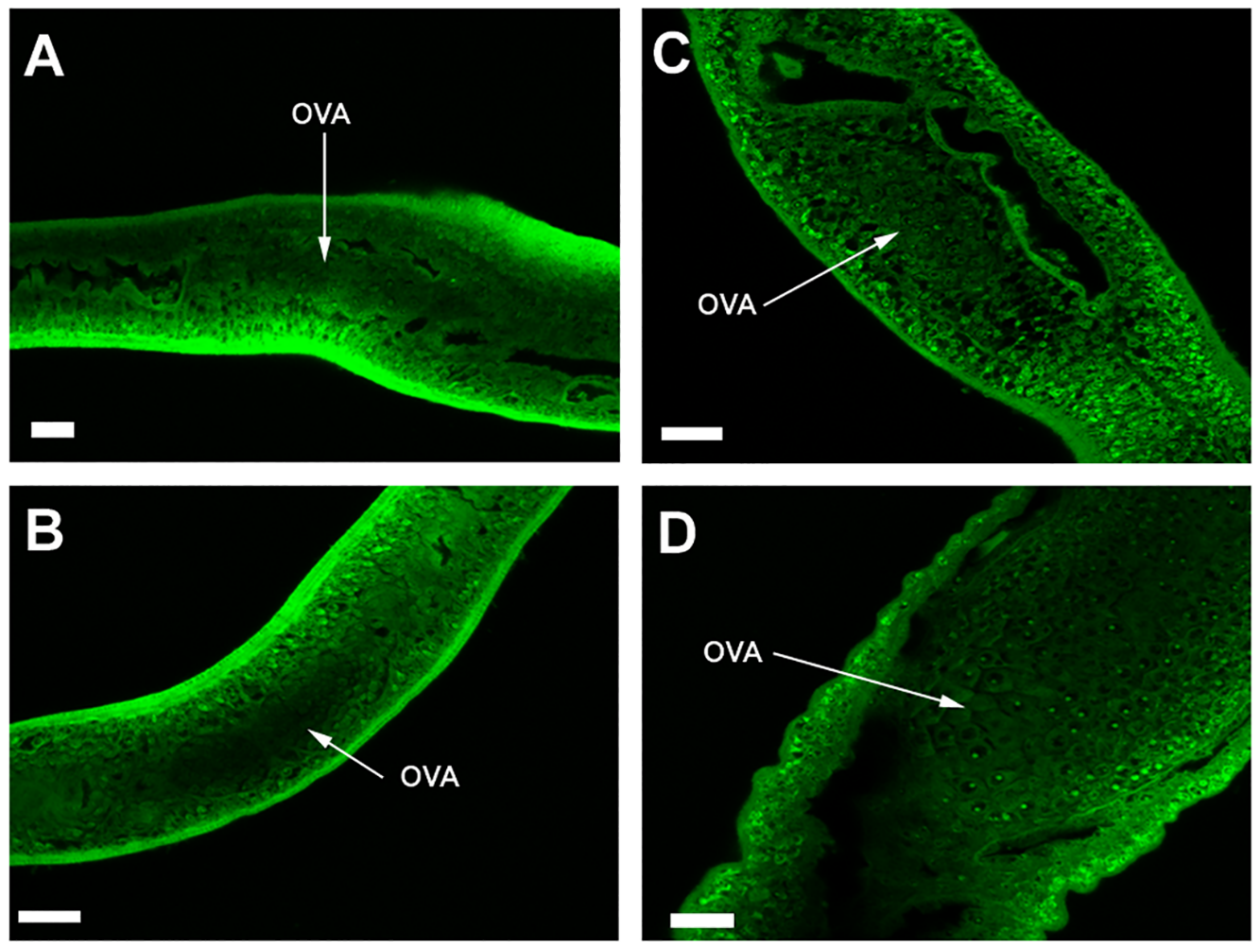
CLSM images of female *S*. *japonicum* from single- and double-sex infections. **A.** 18SSI with a small and incipient ovary.**B.**18DSI with a small and incipient ovary that contains a few oogonia, **C.** 23SSI with an elongated and large ovary that contains a few oogonia. **D.** 23DSI with a mature and large ovary that contains numerous mature and immature oocytes. OVA, ovary; Scale bars, 25 μm.

Through confocal laser scanning microscopy (CLSM), 18DSI and 18SSI were found to possess similar small and incipient ovaries that contain a few oogonia (Fig 1A and 1B). Likewise, they were at similar developmental stages; most females had not paired with males at 18 days post-infection and did not exhibit mature female characteristics. 23SSI was slightly larger in size and possessed an elongated and larger ovary than 18DSI and 18SSI. However, the ovary was still immature and only contained a few oogonia (Fig 1C). Without pairing, 23SSI remained at an immature stage. By contrast, 23DSI not only possessed a mature and bigger ovary than 23SSI, but also contained a considerable amount of mature and immature oocytes (Fig 1D). This phenomenon suggests that pairing of females with males initiated female sex maturation and promoted egg production. Evidently, pairing is vital to the maturation of female sex organs. However, limited information is available on how pairing influences female development via translational regulation.

### Differential Expression of Ribosomal Genes in *S*. *japonicum* Females from Both Single- and Double-Sex Infection at 18 and 23 Days Post-Infection

Results of our Solexa analysis revealed that ribosomal genes are differentially expressed between samples (S1–S4 Tables). Only 9 differentially expressed ribosomal genes were detected between 18DSI and 18SSI (S1 Table), suggesting that these worms possess similar ribosomal gene expression patterns. Similar results, 9 differentially expressed ribosomal genes, were also obtained between 18SSI and 23SSI (S3 Table). However, more differentially expressed ribosomal genes, 30 and 28 genes, were respectively detected between 18DSI and 23DSI or between 23SSI and 23DSI (S2 Table and S4 Table). In particular, most ribosomal genes were upregulated in 23DSI, compared with those in 23SSI. These results indicated that different females at distinct developmental stages have varying gene expression profiles.

**Fig 2:**
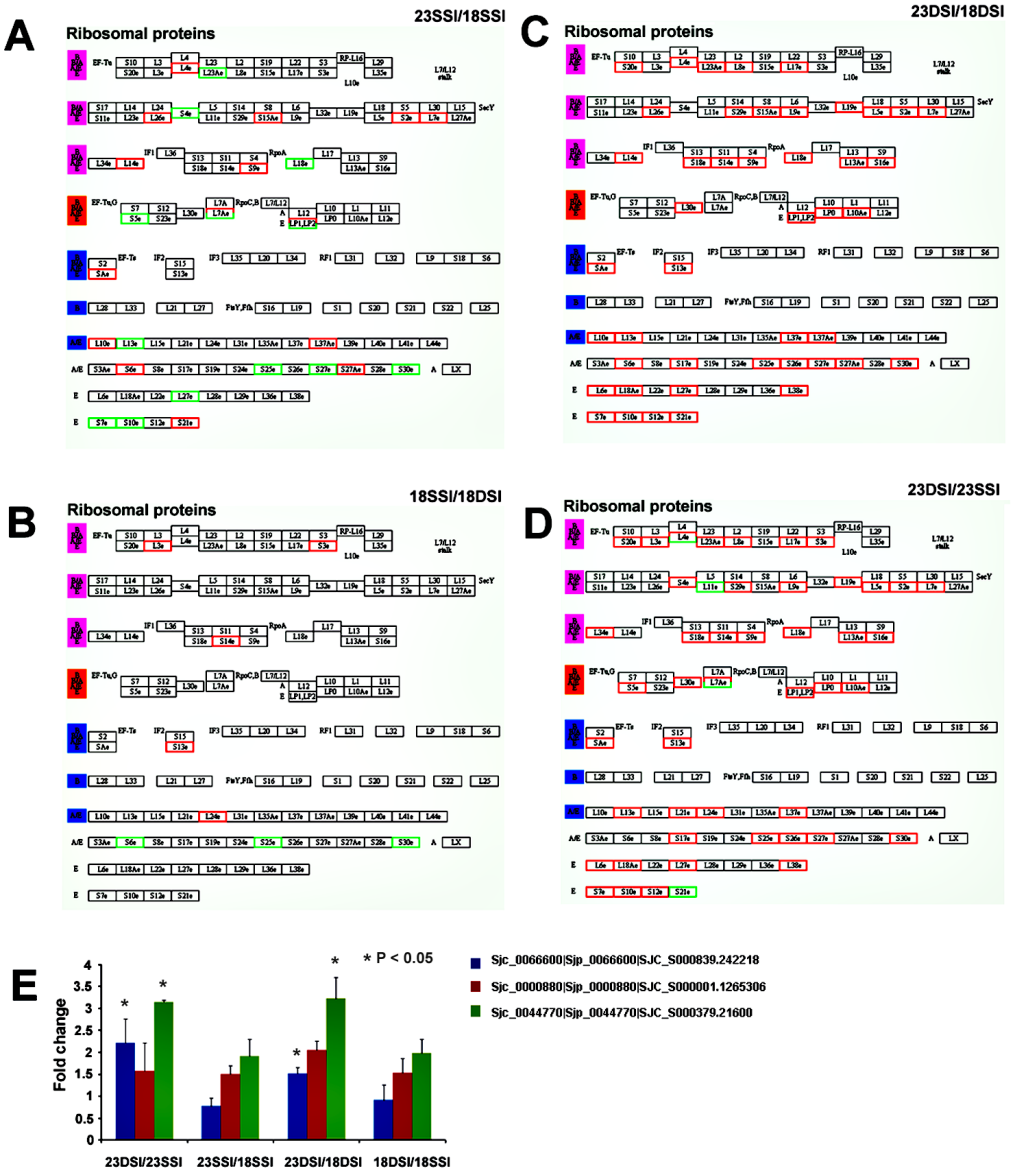
Differential expression of ribosomal genes in female *S*. *japonicum* of single- and double-sex female worms at 18 d and 23 d post-infection. Pathway analysis of differentially expressed ribosomal genes **A.** between 23SSI and 18SSI, **B.** between 18SSI and 18DSI, **C.** between 23DSI and 18DSI, and **D.** between 23DSI and 23SSI.Red, upregulated genes; Green, downregulated genes. **E.** Results of the real-time PCR analysis revealed that the differentially expressed ribosomal genes, such as Sjc_0066600|Sjp_0066600|SJC_S000839.242218,Sjc_0000880|Sjp_0000880|SJC_S000001.1265306,Sjc_0044770|Sjp_0044770|SJC_S000379.21600, showed similar expressions with those found in Solexa analysis. Error bars represent standard deviation obtained from triplicates.(*P<0.05).

The results of pathway analysis of differentially expressed ribosomal genes clearly showed heterogeneous expression profiles of ribosomal genes among 23DSI, 23SSI, 18DSI, and 18SSI (Fig 2A, 2B, 2C and 2D). 18DSI and 18SSI possessed similar expression patterns of ribosomal genes and only a few differentially expressed ribosomal genes were detected between them, consistent with their similar morphologies and developmental stages. However, although 23SSI was larger and older than 18SSI, both worms still shared similar expression profiles of ribosomal genes. It is possible that both 23SSI and 18SSI maintained a similar undeveloped state. By contrast, in 23DSI, not only was the expression pattern of ribosomal genes different but most ribosomal genes were significantly upregulated compared with those in 23SSI and 18DSI. The analysis clearly indicated that the expression patterns of ribosomal genes were more closely associated with developmental stage or degree than with developmental time or age. Results of real-time PCR analysis further confirmed that differentially expressed ribosomal genes in these samples, such as Sjc_0066600|Sjp_0066600|SJC_S000839.242218, Sjc_0000880|Sjp_0000880|SJC_S000001.1265306, Sjc_0044770|Sjp_0044770|SJC_S000379.21600, exhibited expression profiles that were similar to those detected by Solexa analysis (Fig 2E). Interestingly, it was previously reported that most non-ribosomal genes were downregulated in 23DSI compared with 23SSI [3,10]. However, in this study the expression of ribosomal genes in 23DSI exactly violated the rules. Thus, these results imply that these highly expressed ribosomal genes played important roles in sexual maturation of paired females. However, why far more ribosomal genes were upregulated in 23DSI than in 23SSI remains unclear.

### iTRAQ and Solexa Analyses of Differential Gene Expression in 23DSI and 23SSI

**Fig 3:**
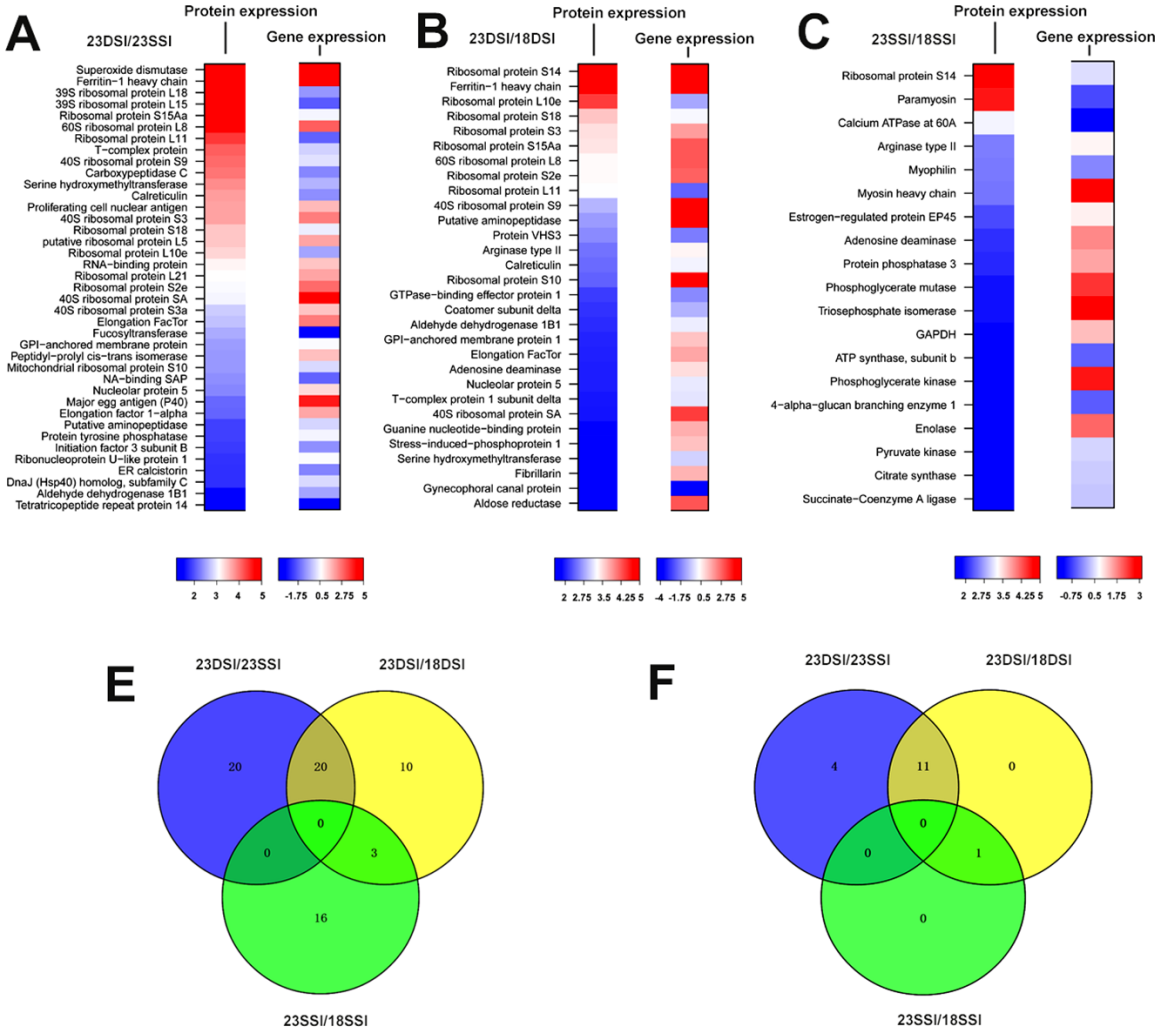
Differentially expressed proteins and genes. **A.** Heat map comparing the translation and transcription of differential proteins between 23DSI and 23SSI by iTRAQ and Solexa analysis. **B.** Between 23DSI and 18DSI. **C.** Between 23SSI and 18SSI. **D.** The quantity of exclusive and common differentially expressed proteins among different samples. **E.** The quantity of exclusive and common differentially expressed ribosomal proteins among different samples. The Venn diagram is created with an online Venn diagram maker. (http://bioinfogp.cnb.csic.es/tools/venny/index.html).

**Fig 4:**
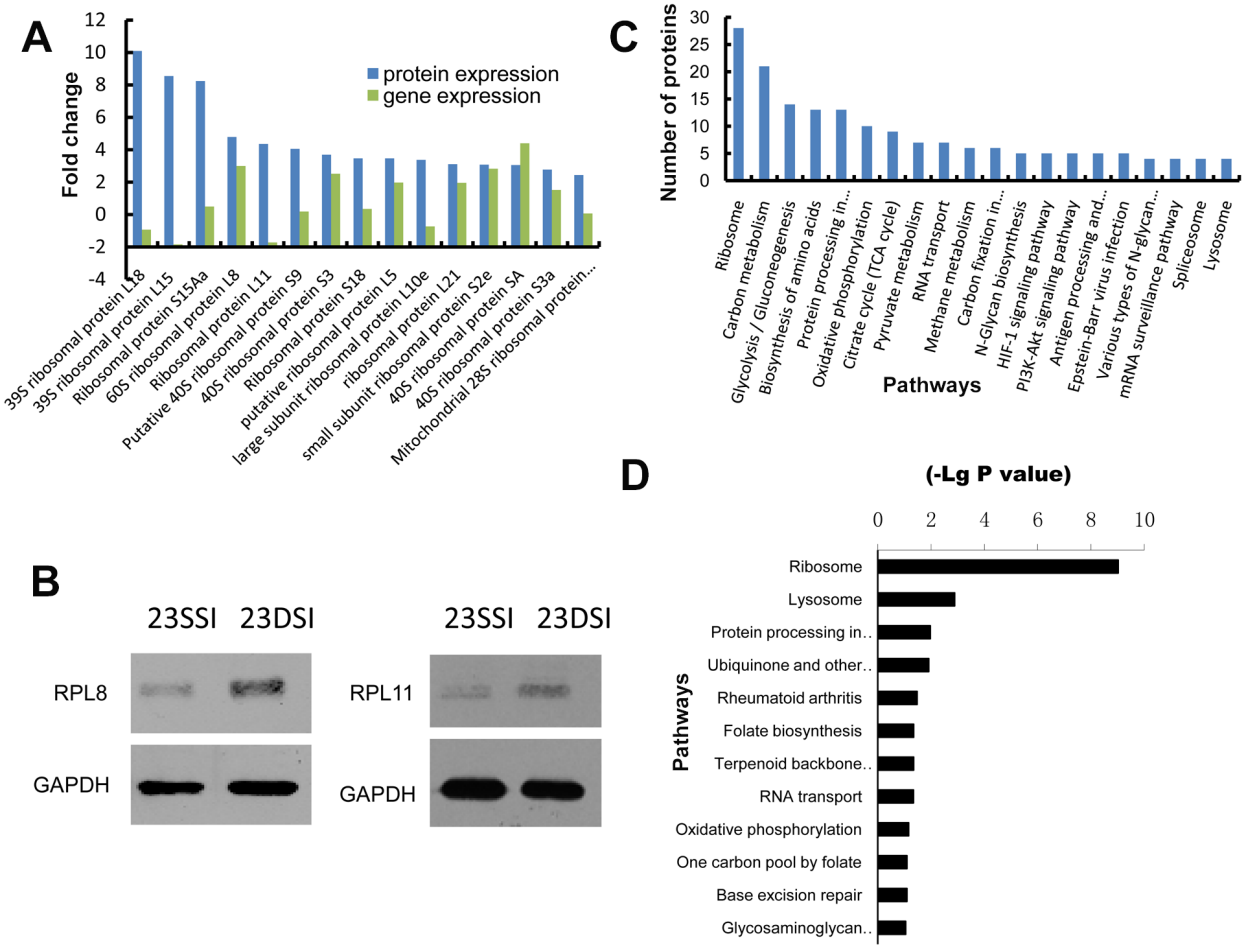
Differential expression of ribosomal proteins and genes between 23DSI and 23SSI and results from KEGG analysis. **A.** Comparison of differentially expressed ribosomal proteins and genes between 23SSI and 23DSI, which indicate that the ribosomal protein translation level is not completely consistent with the gene expression level. **B.** Western blot assay revealed that ribosomal proteins L8 and L11 were highly expressed in 23DSI than in 23SSI. Data are representative of at least 3 individual experiments. **C.** Kegg analysis of differentially expressed proteins between 23SSI and 23DSI. **D.** Kegg analysis of differentially expressed genes between 23SSI and 23DSI.

Since more ribosomal genes were significantly upregulated in 23DSI than in 23SSI, more ribosomes should be assembled, leading to higher levels of protein synthesis of all transcripts in 23DSI. To investigate this assumption, iTRAQ and mass spectrometry were used to analyze the protein profiles of 23DSI and 23SSI. Numerous differentially expressed proteins (234 proteins) were obtained between 23DSI and 23SSI (S5 Table). Interestingly, no elevation in protein synthesis of some upregulated genes was observed in 23DSI compared with 23SSI, based on the analysis of upregulated genes in 23DSI (S6 Table). The profiles of differentially expressed proteins were not in accordance with the profiles of the differentially expressed genes (Fig 3A). For most of the 490 upregulated genes in 23DSI (S6 Table), no corresponding increase in the protein level was detected. Meanwhile, several downregulated genes, or even those not differentially expressed, such as 39S ribosomal protein L18, 39S ribosomal protein L15, ribosomal protein L11, calreticulin, DNA-binding SAP, and carboxypeptidase C were significantly upregulated at the protein level in 23DSI. However, expression of most ribosomal genes actually increased at the protein level (Fig 3A and S7 Table). Although high expression of ribosomal proteins was not completely consistent with gene expression results, more upregulated ribosomal proteins were undoubtedly observed in 23DSI (Fig 4A). Furthermore, western blot results also confirmed the high expression level of ribosomal proteins such as L8 and L11 (Fig 4B). Although it is indisputable that the expression of ribosomal proteins increased in paired females in our study, there seems to contradiction between “more ribosomes” and “less transcripts”. Thus, it is possible that the increase in ribosomal proteins is not for increase in ribosome quantity, but likely for the assembly of novel ribosomes to regulate the expression of necessary proteins. Notably, several mature female-specific proteins, such as superoxide dismutase [Cu-Zn] and ferritin-1 proteins, were also significantly upregulated, which was consistent with their gene expression patterns, in 23DSI (Fig 3A and S7 Table). These results further suggested that specific ribosomal protein profiles or specific ribosome composition could regulate the expression of these mature female-specific proteins.

KEGG analysis of the differentially expressed proteins and genes between 23DSI and 23SSI were performed, respectively. KEGG analysis of the differentially expressed proteins showed that pathways, such as ribosome, carbon metabolism, glycolysis, protein processing in endoplasmic reticulum, oxidative phosphorylation, TCA cycle, RNA transportand lysosome were dominant in 23DSI (Fig 4C). Several pathways identified by KEGG analysis of differentially expressed proteins overlapped with those from KEGG analysis of differentially expressed genes (Fig 4D), such as those involved in protein processing in the endoplasmic reticulum, RNA transport, lysosome, and oxidative phosphorylation. In particular, the ribosome pathway is the most prominent in the two analyses (Fig 4C and 4D). This finding revealed that although the protein profile does not completely correspond to the gene expression profile, for categories such as ribosome, protein processing, and oxidative phosphorylation (which are clearly associated with the development of paired females), both results are consistent. In addition, the results of KEGG analysis of differentially expressed proteins also showed several unique features. For instance, several pathways such as glycolysis/gluconeogenesis, biosynthesis of amino acids, oxidative phosphorylation, and citrate cycle (TCA cycle) dominated in 23DSI. Oxidative phosphorylation and the TCA cycle pathways are considered to be closely associated with fatty acid oxidation and utilization, which are essential for egg production in schistosomes [26–29]. Our results suggest that protein expression not only depends on gene expression, but can more subtly regulate biological functions according to the actual requirements under a given condition. Notably, both analyses showed that ribosome is the most important pathway, suggesting that highly expressed ribosomal proteins should play a vital role in 23DSI.

### Specific Ribosomal Protein Profiles Corresponding to Specific Protein Translation

The similarity in ribosomal gene expression between 18DSI and 18SSI indicated that at the same developmental stage, both had stable and identical ribosomal gene expression profiles, irrespective of whether *S*. *japonicum* came from single- or double-sex infection. Thus, ribosomal protein profiles in 18-day-old females were utilized to compare with those in 23-day-old females to further reveal the relationship between different ribosomal protein profiles and protein translation in *S*. *japonicum*.

**Fig 5:**
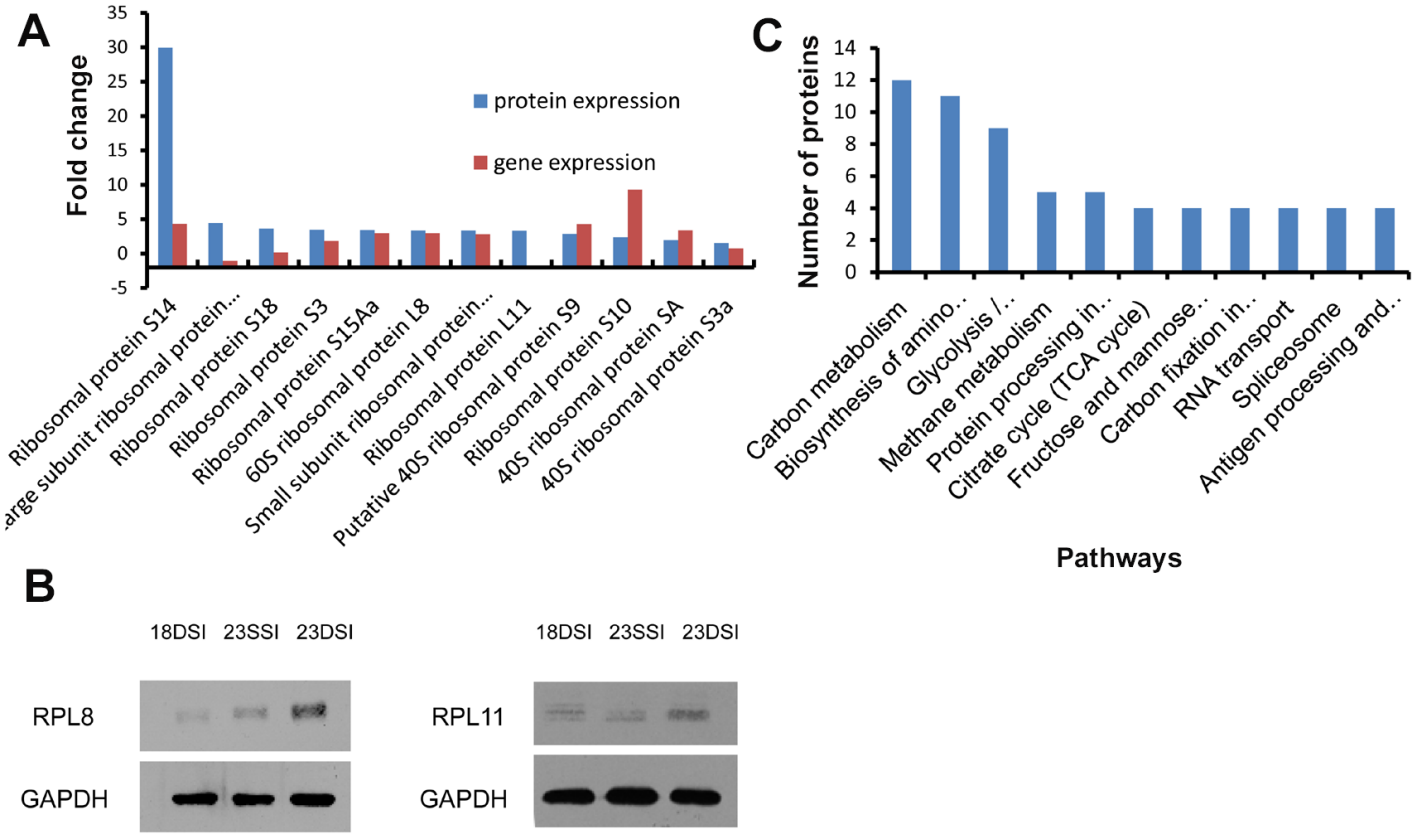
Differential expression of ribosomal proteins and genes, and KEGG analysis. **A.** Comparative results of the differentially expressed ribosomal proteins and genes between 23DSI and 18DSI, which indicate that ribosomal protein translation level is not completely consistent with the gene expression level. **B.** Results of Western blot assay revealed that ribosomal proteins L8 and L11 were highly expressed in 23DSI than in 23SSI and 18DSI. **C.** Kegg analysis of differentially expressed proteins between 23SSI and 18SSI.

From 18DSI to 23DSI or from 18SSI to 23SSI, the former underwent the pairing process, which promoted female sex maturation, whereas the latter did not. Comparative analysis between 23DSI and 18DSI also showed that more ribosomal genes, such as 60S ribosomal protein L8 and ribosomal protein L11, were transcribed and translated in 23DSI, although several of them were downregulated at the transcript level (Figs 3B and 5A). The results of Western blot analysis further confirmed that several proteins, such as 60S ribosomal protein L8 and ribosomal protein L11, are highly expressed in 23DSI compared with that in 18DSI (Fig 5B). In addition, several mature female-specific proteins, such as ferritin-1 heavy chain, were significantly upregulated at the transcription and translation levels in 23DSI (Fig 3B and S8 Table). Notably, Solexa analysis detected 705 upregulated genes in 23DSI (S9 Table), but only 156 proteins were upregulated. Obviously, no corresponding increase in the protein level was detected (S10 Table). This result indicated that the higher number of highly-expressed ribosome proteins in 23DSI only enhance translation of some mature female-specific transcripts. Evidently, the difference in protein profiles between 18DSI and 23DSI was similar to that between 23SSI and 23DSI, particularly in differentially expressed ribosomal proteins and mature female-specific proteins. This similarity was likely caused by the similar ribosomal protein expression patterns between 23SSI and 18DSI. Although 23SSI was older than 18DSI, both of them were unpaired females and still possessed similar ribosomal protein expression patterns. Thus, the result also supports the idea that pairing regulates ribosomal protein expression patterns.

By contrast, hardly any ribosomal proteins were found to be differentially expressed between 18SSI and 23SSI (Fig 3C and 3E, S11 Table), except for ribosomal protein S14. Moreover, the number of differentially expressed proteins was far smaller than that between 18DSI and 23DSI (Fig 3D, S10 Table and S12 Table). This suggested that 18SSI and 23SSI possessed similar ribosome quantities or compositions, which led to similar protein profiles. Several mature female-specific protein genes, such as ferritin-1 heavy chain gene, were found to be expressed in 23SSI. However, no evidence of elevated expression of ferritin-1 heavy chain was found at the protein level, suggesting that the ribosomal protein profile in 23SSI did not facilitate the expression of these female-specific proteins. In addition, results of KEGG analysis revealed several proteins that were differentially expressed between 18SSI and 23SSI; these proteins were involved in many pathways such as carbon metabolism, biosynthesis of amino acids, glycolysis/gluconeogenesis, methane metabolism, protein processing in endoplasmic reticulum, and TCA cycle (Fig 5C). In particular, several specific pathways such as the ribosome and oxidative phosphorylation pathways were found not to be involved, suggesting that expression of ribosomal proteins was not essential in unpaired females. As a result, only less differentially expressed proteins were found between 18SSI and 23SSI. Moreover, hardly any mature female-specific proteins were detected, suggesting that neither the extension of development time nor the increase of worm size could not essentially “change” the protein profile. The differential expression of mature female-specific proteins was likely determined by differential expression of ribosomal proteins. These results further indicate that the differential expression of numerous ribosome proteins after pairing is essential for female sex maturation.

### Simultaneously Expressed Genes Sharing the Common Motif

**Fig 6:**
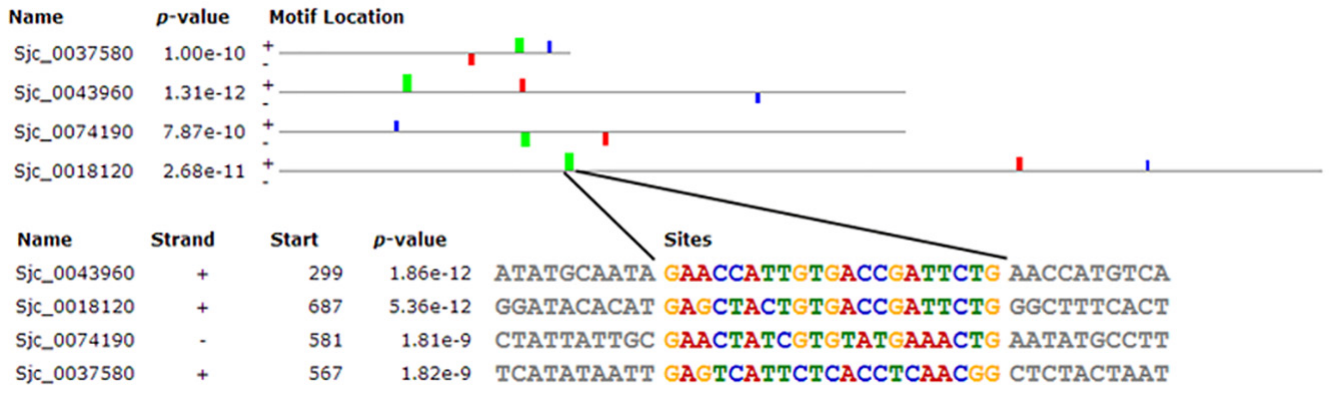
Motif analysis of 4 downregulated ribosomal genes in 23DSI. All of 3 motifs appear in these genes. The sequence and site of one motif are showed.

**Fig 7:**
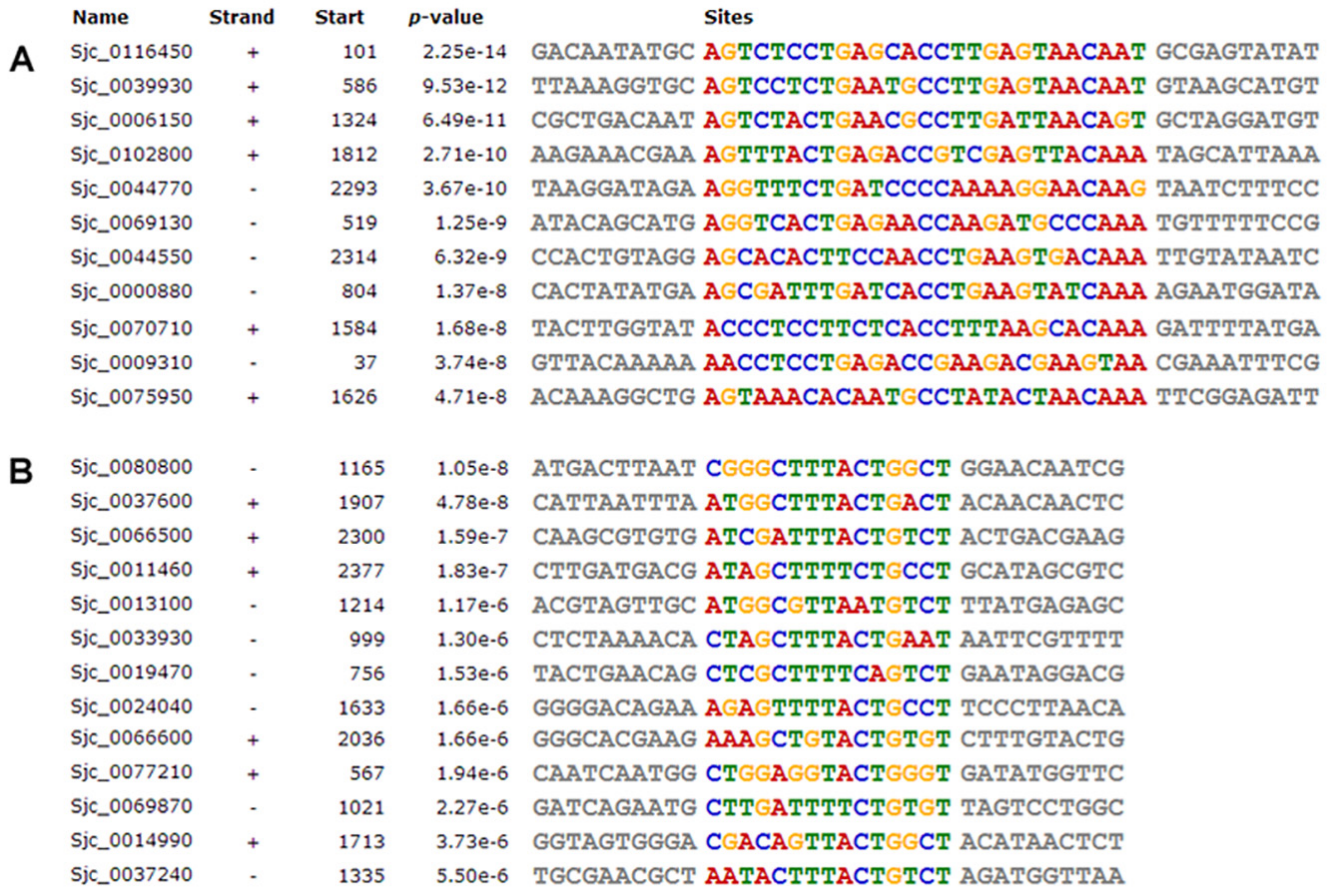
Motif analysis of 24 upregulated ribosomal genes in 23DSI. **A.** A common motif was found in 11 upregulated ribosomal genes. **B.** Another motif in other upregulated ribosomal genes. The sequence and site of the two motifs are showed.

Compared with 23SSI, the motifs of differentially expressed ribosomal genes were analyzed in 23DSI (S4 Table). Based on the available data from *Schistosoma japonicum* contig about ribosomal genes, 28 promoter sequences were obtained. Of them, there were 24 promoter sequences of upregulated ribosomal genes. MEME analysis of their promoter sequences identified 3 common motifs enriched in the downregulated genes (Fig 6) and 2 common motifs in two different subgroups of upregulated ribosomal gene, respectively (Fig 7). In subgroup A, 11 of 24 upregulated ribosomal genes shared a common motif (Fig 7A), whereas in subgroup B, 13 of 24 genes shared another common motif (Fig 7B). The downregulated and upregulated ribosomal genes shared the common motifs, suggesting that their expression be regulated by the same factors. These results provide a better understanding of why these genes are simultaneously expressed or inhibited.

Ribosomes are important players in the growth and development of cells. Any defect in ribosomal components or any loss of core ribosomal proteins can alter gene expression patterns [30,31], which can result in tissue-specific changes. Some ribosomal proteins have several extra ribosomal functions, such as apoptosis induction [32,33], DNA repair [34,35] and development regulation [36–41]. In addition, overexpression or depletion of several ribosomal proteins have been shown to suppress or upregulate gene transcription [42]. Several ribosomal proteins can bind to specific structures within mRNAs to control expression of certain mRNA species [43]. Some ribosomal proteins can even regulate their own pre-mRNAs to modulate their expression through a feedback mechanism [44]. Thus, in addition to protein synthesis, dynamic and heterogeneous expression patterns of ribosomal proteins likely play important roles in protein translation regulation.

In the present study, 23DSI, the paired female *S*. *japonicum* were found to possess numerous highly expressed ribosomal genes, and its specific protein profiles were different from those of unpaired females such as 23SSI and 18DSI. Notably, no changes in overall protein synthesis were observed despite elevated expression of diverse ribosomal proteins in 23DSI, with the exception of some mature female-specific proteins that showed elevated expression. This implies that a specialized ribosomal protein profile in paired females may meet the specific demands of sexual maturation after pairing. Besides, upregulation of some ribosomal proteins likely promote functional specialization of the original ribosome by regulating ribosomal heterogeneity through processes that increase several key protein components and change the protein modification state, as previously reported in other organisms [12].

Taken together, our findings revealed that differential expression of ribosomal proteins before and after pairing, implying that the specialized ribosomal protein expression profiles or the assembly of specialized ribosomes is responsible for the regulation of the translation of specific proteins involved in *S*. *japonicum* sexual maturation.

## Supporting Information

S1 Table Differentially expressed ribosomal genes between 18DSI and 18SSI. (XLSX)

S2 Table Differentially expressed ribosomal genes between 18DSI and 23DSI. (XLSX)

S3 Table Differentially expressed ribosomal genes between 18SSI and 23SSI. (XLSX)

S4 Table Differentially expressed ribosomal genes between 23SSI and 23DSI. (XLSX)

S5 Table Differentially expressed proteins between 23DSI and 23SSI based on iTRAQ analysis. (XLSX)

S6 Table Upregulated genes in 23DSI compared with 23SSI based on Solexa analysis. (XLSX)

S7 Table Comparison between translation and transcription of differential proteins between 23DSI and 23SSI by iTRAQ and Solexa analysis. (XLSX)

S8 Table Comparison between translation and transcription of differentially expressed proteins between 23DSI and 18DSI by iTRAQ and Solexa analysis. (XLSX)

S9 Table Upregulated genes in 23SSI compared with 23DSI based on Solexa analysis. (XLSX)

S10 Table Differentially expressed proteins between 18DSI and 23DSI based on iTRAQ analysis. (XLSX)

S11 Table Comparison between translation and transcription of differential proteins between 23SSI and 18SSI by iTRAQ and Solexa analysis. (XLSX)

S12 Table Differentially expressed proteins between 18SSI and 23SSI based on iTRAQ analysis. (XLSX)
